# Adverse pregnancy outcomes in rural Maharashtra, India (2008–09): a retrospective cohort study

**DOI:** 10.1186/1471-2458-12-543

**Published:** 2012-07-23

**Authors:** Prakash Prabhakarrao Doke, Madhusudan Vamanrao Karantaki, Shailesh Rajaram Deshpande

**Affiliations:** 1 Community Medicine Department, MGM Medical College, Kamothe, Navi Mumbai 410 210, India; 2 State Family Welfare Bureau, Government of Maharashtra, Pune, 411 001, India; 3 Symbiosis School of Biomedical Sciences, Lavale, Taluka; Mulshi, District, Pune, 412 005, India

**Keywords:** Relative risk, Abortion, Stillbirth, Sex ratio at birth, Tribal areas

## Abstract

**Background:**

The study was carried out to record adverse pregnancy outcomes and to obtain information about sex ratio at birth in rural especially tribal areas in the State of Maharashtra, India. Although the tribal population is considered vulnerable to innumerable adversities, regretfully information about pregnancy wastage among them is not available. About 10% population of the state is tribal. The study of sex ratio at birth was planned as the overall sex ratio and child sex ratio had declined in the state.

**Methods:**

The cohort of antenatal cases registered in rural areas of Maharashtra in the calendar year 2008 was followed up to study the pregnancy outcomes. A retrospective study was carried out from October 2009 to August 2010. The outcomes of all the registered antenatal cases were recorded by the Auxiliary Nurse Midwives. The summary sheets were obtained by Block Medical Officers. The data was entered at the block level by trained data entry operators in specially designed web-based software. Adverse pregnancy outcome was categorized in two groups abortions and stillbirths.

**Results:**

About 1.1 million registered pregnancies were followed up. In the state 5.34% registered pregnancies ended in abortions. In tribal PHCs the relative risk of spontaneous abortion and induced abortion was 0.91and 0.38 respectively. It was also revealed that about 1.55% pregnancies culminated in stillbirth. The relative risk of stillbirths in tribal PHCs was 1.33. The sex ratio at birth in the state was 850. The ratio was 883 in the tribal PHCs. Correlation was observed between sex ratio at birth and induced abortion rate.

**Conclusions:**

The study indicates that women from tribal PHCs are exposed to higher risk of adverse pregnancy outcome in the form of stillbirths. In non-tribal areas high induced abortion rate and poor sex ratio at birth is observed. These two indicators are correlated. The correlation may be explained by the unscrupulous practice of sex selective abortion.

## Background

The Government of India in the year 2005 launched the National Rural Health Mission (NRHM), for a period of seven years aiming at reduction of Infant Mortality Rate (IMR), Maternal Mortality Ratio (MMR) and Total Fertility Rate (TFR). The NRHM has already completed seven years of implementation.

The quality of maternal health services is a determinant for all the goals stated above. It was decided to study pregnancy outcome, which serves as a proxy to the quality of maternal health care services. While deliberating infant mortality, neonatal deaths receive sufficient focus however stillbirths are not taken into account adequately. Although reports from India vary, [1-3] global estimates indicate that early neonatal deaths and stillbirths are of equal magnitude. [4-6] In India estimates are available from the Sample Registration System (SRS), National Family Health Survey (NFHS) and District Level Household Survey (DLHS) data. The Health Management Information System (HMIS) data of the Health Department, Government of Maharashtra also provides such information. All above sources do not provide information about tribal areas while SRS and NFHS do not provide even district data. Vulnerability of tribal people is well documented [7]. The NRHM envisages special action plan for tribal areas. We while working under NRHM, Government of Maharashtra intended to study adverse pregnancy outcomes and relative risk in tribal areas. In the state of Maharashtra, both the sex ratio and child sex ratio have declined in the decade 1991–2001, and therefore it was felt imperative to find out recent status of sex ratio at birth. The study was conducted utilizing available funds.

## Methods

The study was a retrospective cohort study carried out in the State of Maharashtra, which is the second most populous state in India. As per the last census carried out in 2011, the population is 112.378 million, of which 45.23% is urban and 54.77% rural. As per 2001 census 8.85% population is tribal. Tribal people are believed to be aboriginal population of India. They inhabit in cloistered, remote areas such as hills and forests. Their livelihood is dependent on archaic farming and inherent naïve products like honey, Tendu leaves etc. Their culture, language and religion are distinctive. They customarily do not socialize with dissimilar urbanity and population. As a result they are economically weak and have low levels of literacy and health. In the state there are 35 districts inclusive 2 from, Mumbai Municipal Corporation. The rural including tribal population is scattered across remaining 33 districts. The 15 districts which have substantial number of tribal population (presently minimum 0.2 million in each district) are notified as tribal districts. Even in these districts the tribal population mainly lives in few blocks. There are 1,816 Primary Health Centers (PHCs), out of which 320 are located in tribal pockets in these 15 districts. These 320 PHCs are designated as tribal PHCs. Each PHC has about six sub centers. An Auxiliary Nurse Midwife (ANM) manages one sub center. She records all antenatal cases in a formatted register. Only pregnant women who are residents of the sub center area are serially enlisted in the register and are provided services. Pregnant women who are not residents of the sub center area are provided services without numbering. The PHC wise data is entered online for HMIS on monthly basis at block level.

The cohort of 1,183,312 already registered antenatal cases in the calendar year 2008 (information from HMIS) was selected as the study population. The study started from October 2009. First, all the ANMs were instructed to write all the details of the women including the follow up to 42 days after abortion or delivery during their home visits. After completing the data collection a summary sheet was submitted to Block Medical Officer through Medical Officer of PHC. A separate link on the website of the Directorate of Health Services, Government of Maharashtra was created to enter the new data. Training of the concerned staff was undertaken in three phases for obtaining good quality data. In first phase all the Block Medical Officers and the block level data entry operators were trained at the divisional level by the investigators. Block level personnel trained supervisors from PHCs. In the third phase ANMs were trained. For each PHC sub center wise data entry was carried out at the block level. The data entry was completed by August 2010. The data was cross checked with available HMIS data. Nil reports in any outcome and more than double the expectations were returned. The concerned ANM was once again briefed about the study and resubmission was made obligatory.

Pregnancy outcome was categorized as abortion, stillbirth and live birth regardless of multiple pregnancies. The investigators calculated the relative risk of abortion and stillbirth. Sex ratio at birth was also calculated.

The term abortion is defined as termination of pregnancy before 28 weeks of gestation.

Abortion rate is defined as number of abortions per 100 pregnancies.

Stillbirth is defined as per World Health Organization standards, "delivery of a baby after 28 weeks of gestation, but which did not show any signs of life like crying, respiration, heartbeat or any movements", for international comparison [8].

Stillbirth rate is defined as number of stillbirths per 100 pregnancies.

Sex ratio is defined as number of females per 1000 males in the population.

Child Sex-ratio has been defined as the number of females in age-group 0–6 years per 1000 males in the same age-group in the population.

Sex ratio at birth is defined as the number of girls born alive per 1,000 boys born alive.

The study was approved and ethical clearance was given by the Institutional Committee of State Health System Resource Center, Maharashtra State.

## Results

We studied pregnancy outcomes of 1,070,154 pregnancies in rural area of the Maharashtra State. About 9.6% women were lost to follow up either due to vacancy of ANMs or non-availability of the women in their houses. Table1 shows comparison between tribal and non-tribal PHCs. The abortion rate was higher in non-tribal PHCs and stillbirth rate was higher in tribal PHCs (*χ*^2^ = 1913.11, P < 0.0001). The relative risk of spontaneous abortion was 0.91 and induced abortion was 0.38 in tribal PHCs, whereas the relative risk of stillbirths was 1.33. The sex ratio in tribal PHCs was better than non-tribal PHCs (*χ*^2^ = 73, P < 0.001).

**Table 1:** Outcomes of pregnancy in tribal and non-tribal PHCs of Maharashtra, India (2008–09)

**Area**	**Pregnancies followed**	**Abortions**			**Stillbirths*(%)***	**Live births**			
		*Spontan. (%)*	*Induced (%)*	*Total (%)*		*Male*	*Female*	*Total (%)*	SRB*
**Tribal PHCs**	174,249	4,776 (2.74)	1,744 (1.00)	6,520 (3.74)	3,416 (1.96)	87,262	77,051	164,313 (94.30)	883
**Non- Tribal PHCs**	895,905	26,845 (3.00)	23,8 0 (2.66)	5,06 5 (5.66)	13,148 (1.47)	451,478	380,634	832,112 (92.88)	843
**Total**	1,070,154	31,621 (2.95)	25,544 (2.39)	57,165 (5.34)	16,564 (1.55)	538,740	457,685	996,425 (93.11)	850

*SRB = Sex Ratio at Birth.

**Figure 1:**
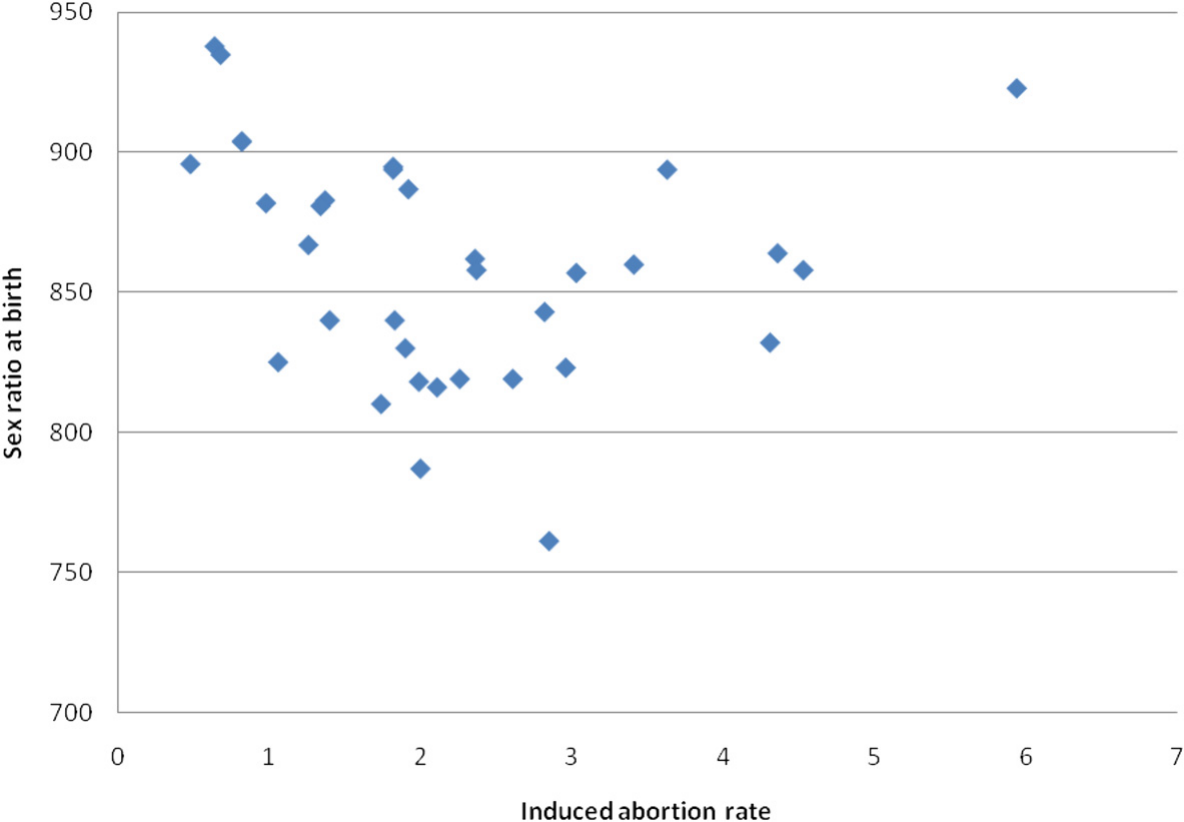
Induced abortion rate and sex ratio at birth in the districts of Maharashtra, India.

Table2 gives of interquartile range of the outcomes. The highest figure of spontaneous abortion rate was 4.1 times the lowest figure. In the last quartile having high spontaneous abortion rate there were only two tribal districts. Variation in the statistics of induced abortion rate is very wide. The highest figure was 12.4 times the lowest figure. In the quartile recording lesser rates, there were six tribal districts and last three were all predominantly tribal districts. The last quartile of districts having high induced abortion rate comprised six non-tribal districts including two coastal districts of Sindhudurg and Ratnagiri. The highest stillbirth rate was 3.3 times the lowest rate. The high rate quartile included six tribal districts and the first three districts were predominantly tribal. Out of the eight districts in quartile recording higher sex ratio, four were tribal districts including the best two. The two coastal districts were also in that quartile. Excluding the two coastal districts Sindhudurg and Ratnagiri the correlation coefficient between induced abortion and sex ratio at birth was 0.42 (P < 0.02) and depicted in Figure1.

**Table 2:** Outcomes of pregnancy in districts of Maharashtra, India (2008–09)

**S. No.**	**Outcome**	**Interquartile range**
1	Spontaneous abortion rate	2.09-3.51%
2	Induced abortion rate	1.35-2.95%
3	Stillbirth rate	1.31-1.89%
4	Sex ratio at birth	824-891

## Discussion

About 25% pregnancies result in abortions either spontaneous or induced. About 75% of the abortions occur before 16–20 weeks of gestation and out of these abortions, 75% occur before eight weeks of gestation [9]. Abortion rate in the State of Maharashtra was found to be 7.6%, in the DLHS-3 carried out in 2007–08. The same survey recorded 61.6% pregnancies were registered in the first trimester of gestation [10]; meaning that 38.4% pregnancies were registered after the maximum risk of abortion was almost over. We observed 5.34% pregnancies ended in abortions indicating high reliability of data. The reports from DLHS-3 indicated that in the country 6.5% pregnancies end in abortions [11]. Our study recorded that 44.76% abortions were induced. The percentage of induced abortions in state in DLHS 3 was 36.84% [10]. At the national level 28.92% abortions were induced [11]. The segregated data shows that 26.74% and 46.99% abortions are induced in tribal and non-tribal tribal PHCs respectively. The proportion of induced abortions is higher in non-tribal PHCs (*χ*^2^ = 957.03, P < 0.0001). This may be due to abundant availability of abortion services in the state, particularly in non-tribal areas, and social factors like literacy, economic status etc. It is estimated that in India, more than 6 million abortions are induced and only 10% are officially reported [12]. Substantial percentage of induced abortions is suspected to be sex selective [12]. In most part of the world the reported data about abortions are usually underestimates [13,14]. As unsafe abortions are a threat to women’s health and survival, many studies attempt to estimate safe/unsafe abortions or legal/illegal abortions. Worldwide abortion rate range from 12 to 44 per 1,000 women aged 15–44 years [13-15].We did not differentiate between legally or illegally induced abortions to obtain precise information.

A study carried out by one voluntary organization in Maharashtra recorded still birth rate of 31.5/1,000 births for the period 1998–2000 [16]. In 2005–06 a study carried out in the slums of Mumbai city recorded still birth rate of 16.5/1000 births [17]. Field based rural studies in the country reported stillbirth rates from 13.5 to 31.8 per 1,000 births [18-21]. Latest DLHS-3 recorded stillbirth rate of 1.4% in Maharashtra [10]. The findings of present study closely match Mumbai study and DLHS-3. In India, reports from the SRS in the current millennium show that the stillbirth rate is almost constant at about 8-9/1,000 births. This estimate is the lowest among national estimates [1,2]. The NFHS 3 and DLHS 3 recorded stillbirth rates between 1.92 and 1.3/100 pregnancies in the country, [11] and both the periodical surveys have shown some decline over previous rounds. In the WHO estimates, stillbirth rate of India is shown as 39/1,000 births [4]. In the recent estimate India ranks 1st in absolute number and the estimated rate is about 30 [22]. From neighboring Bangladesh, a study recorded stillbirth rate of 30.74/1,000 births [23].In the slum areas of Karachi in Pakistan the stillbirth rate was 27/1,000 births [6]. The overwhelming majority (98%) of stillbirths occur in low/middle income countries [4,5,23]. Recent multi-centric studies have shown stillbirth rates of 12.5-23.0/1,000 births in developing countries [5,24]. In developed countries, the reported stillbirth rate is about 5-8/1,000 births [15,18]. Globally out of estimated 3.2-3.3 million stillbirths for year 2000, only a small fraction, about 2% are registered [25,26]. Stillbirths are grossly underreported in the developing countries [4,16,27]. The latest estimate (2009) of 2.64 million stillbirths across the world has shown decline [22]. The vast majority of stillbirths are preventable and there is immense need to enumerate them [28]. Enumeration is the first step in analysis and then prevention. From public health perspective, there is utmost need of meticulous information about period of stillbirths i.e. whether ante-partum or intra-partum, associated conditions, underlying causes and availability and quality of health care.

Considering the high risk of stillbirths in tribal areas due emphasis needs to be given on availability and quality of ante-natal and obstetric care. Another reason for attention to stillbirth is its significant association with maternal mortality [29].

Sex ratio at birth gives latest indication of female male proportion. In a country wide study, the adjusted sex ratio for second birth when the preceding child was a girl was 759 per 1,000 males. The adjusted sex ratio for the third child was 719 if the previous two children were girls [30]. The documented sex ratio at birth in the present study was poorer than observed in a rural area of south India [19], and reported in SRS for the years 2006–2008 for the state [31]. The sex ratio at birth was lowest in Beed district. This is consistent with lowest child sex ratio reported in an earlier study mapping latest child sex ratio in the state [32]. We attempted to study the correlation of sex ratio at birth with induced abortion rate. Among top 50% i.e. 16 districts recording high induced abortion rate (more than 2%), all districts excluding two have poor sex ratio of 864 or less at birth. The two districts are Sindhudurg and Ratnagiri. These districts have high induced abortion rate but have better sex ratio at birth (>894). The districts are from the western coastal division of Maharashtra, in which Mumbai is located. A sizable proportion of men in the productive age group from these districts are working in and around Mumbai. This is reflected even in the 2011census in two forms. Firstly, both Mumbai districts and these two districts are ranked lowest four in the state with regard to proportion of children in 0–6 year age group. Secondly best overall sex ratio is observed in these two districts whereas lowest sex ratio in two districts of Mumbai [33]. A study has vividly shown that among the migrants in Mumbai the highest number are from these two districts [34]. Pregnancy in a woman whose husband is away from home is culturally not acceptable in the society and hence terminated. In fact, these two districts are constituted by bifurcation of one district. The correlation therefore was calculated excluding these districts. We observed correlation although not strong but certainly significant. Earlier in a study carried out in the state, a clear association was revealed between the sex ratio and number of ultrasonography centers [35]. The present study reveals that there is a negative correlation between induced abortion rate and sex ratio at birth. This clearly points to the fact that sex selective abortions are being vehemently practiced in the districts in spite of the Pre-conception and Pre-natal Diagnostic Techniques (PCPNDT) Act, which provides deterrent punishment for sex determination [12,17]. The alarmingly low sex ratio at birth observed in this study should be a guiding factor to strengthen the steps against sex selective elimination in the state, especially in areas with high number of ultrasonography centers and high induced abortion rate. The better sex ratio at birth in tribal areas may be due to non-availability, non-affordability of the diagnostic techniques and also can be attributed to cultural and ethnic beliefs.

### Limitations of the study

Physical verification of stillbirth/abortion could not be carried out as it was a retrospective study. Further in spite of imparting training to ANMs, some extent of the inherent misclassification of abortion, stillbirth and early neonatal death may have remained in the study.

## Conclusions

In areas where tribal population is substantial separate data analysis and activities accordingly need be carried out to achieve the targeted goals under national programs. The study indicates that tribal women are exposed to higher risk of adverse pregnancy outcome in the form of stillbirths. Therefore basic emergency obstetric care requires to be provided in tribal areas on a priority. In non-tribal areas high induced abortion rate and poor sex ratio at birth is observed. These two indicators are correlated. This indicates that sex selective abortion is practiced. The data generated indicates that accurate information can be obtained independent to the Management Information System data. This system seems to be replicable.

## Competing interests

The author(s) declare that they have no competing interests.

## Authors’ contribution

Doke PP initiated the concept of the study and finalized the design, participated in training of the block level personnel and followed the field level personnel for obtaining the data. Karnataki MV assisted in finalizing the concept, persuaded for acquisition of data and carried out maximum training. Deshpande SR assisted in finalization of design, participated in training and assisted in manuscript drafting. All authors read and approved the final manuscript.

## Pre-publication history

The pre-publication history for this paper can be accessed here:

http://www.biomedcentral.com/1471-2458/12/543/prepub
